# Low-Level Light Therapy Protects Red Blood Cells Against Oxidative Stress and Hemolysis During Extracorporeal Circulation

**DOI:** 10.3389/fphys.2018.00647

**Published:** 2018-05-31

**Authors:** Tomasz Walski, Anna Drohomirecka, Jolanta Bujok, Albert Czerski, Grzegorz Wąż, Natalia Trochanowska-Pauk, Michał Gorczykowski, Romuald Cichoń, Małgorzata Komorowska

**Affiliations:** ^1^Department of Biomedical Engineering, Faculty of Fundamental Problems of Technology, Wrocław University of Science and Technology, Wrocław, Poland; ^2^Regional Specialist Hospital in Wrocław, Research and Development Centre, Wrocław, Poland; ^3^Institute of Cardiology, Warsaw, Poland; ^4^Department of Animal Physiology and Biostructure, Faculty of Veterinary Medicine, Wrocław University of Environmental and Life Sciences, Wrocław, Poland; ^5^Medinet Heart Center Ltd., Wrocław, Poland; ^6^Department of Internal Medicine and Clinic of Diseases of Horses, Dogs and Cats, Faculty of Veterinary Medicine, Wrocław University of Environmental and Life Sciences, Wrocław, Poland

**Keywords:** cardiopulmonary bypass (CPB), low-level light therapy (LLLT), red blood cell, extracorporeal circulation (ECC), near-infrared radiation (NIR), heart-lung machine, hemolysis, photobiomodulation

## Abstract

**Aim:** An activation of non-specific inflammatory response, coagulation disorder, and blood morphotic elements damage are the main side effects of the extracorporeal circulation (ECC). Red-to-near-infrared radiation (R/NIR) is thought to be capable of stabilizing red blood cell (RBC) membrane through increasing its resistance to destructive factors. We focused on the development of a method using low-level light therapy (LLLT) in the spectral range of R/NIR which could reduce blood trauma caused by the heart-lung machine during surgery.

**Methods:** R/NIR emitter was adjusted in terms of geometry and optics to ECC circuit. The method of extracorporeal blood photobiomodulation was tested during *in vivo* experiments in an animal, porcine model (1 h of ECC plus 23 h of animal observation). A total of 24 sows weighing 90–100 kg were divided into two equal groups: control one and LLLT. Blood samples were taken during the experiment to determine changes in blood morphology [RBC and white blood cell (WBC) counts, hemoglobin (Hgb)], indicators of hemolysis [plasma-free hemoglobin (PFHgb), serum bilirubin concentration, serum lactate dehydrogenase (LDH) activity], and oxidative stress markers [thiobarbituric acid reactive substances (TBARS) concentration, total antioxidant capacity (TAC)].

**Results:** In the control group, a rapid systemic decrease in WBC count during ECC was accompanied by a significant increase in RBC membrane lipids peroxidation, while in the LLLT group the number of WBC and TBARS concentration both remained relatively constant, indicating limitation of the inflammatory process. These results were consistent with the change in the hemolysis markers like PFHgb, LDH, and serum bilirubin concentration, which were significantly reduced in LLLT group. No differences in TAC, RBC count, and Hgb concentration were detected.

**Conclusion:** We presented the applicability of the LLLT with R/NIR radiation to blood trauma reduction during ECC.

## Introduction

Extracorporeal circulation (ECC) is a procedure routinely used in cardiac surgery (e.g., in coronary artery bypass grafting, valve surgery). During the procedures utilizing cardiopulmonary bypass (CPB) an extravasated blood comes into contact with the polymer surfaces of oxygenators, catheters, cannulas, pumps, and filters. It results in non-specific inflammatory response, destruction of blood cells, coagulation activation, and increase in oxidative stress. These factors may evoke a systemic inflammatory response syndrome which contributes to the morbidity and mortality of patients undergoing cardiac surgery (Hirai, 2003).

Hemolysis is one of the common problems occurring in the course of ECC but still requiring solution, as damaged red blood cells (RBCs) may induce toxic effects (Vercaemst, 2008). Even low concentrations of plasma-free hemoglobin (PFHgb) contribute to a significant increase in RBC aggregation in the presence of small shear stress (Ji and Undar, 2006; Cabrales, 2007). This, in turn, causes an increase in blood viscosity and thus a high resistance in capillaries. PFHgb also binds nitric oxide (NO) at the level of microcirculation after depletion of haptoglobin (Hp) stores (Cabrales, 2007). As a consequence, tissue hypoxia, in the presence of already reduced RBC count, is escalating.

Moreover, transfusion of RBC is associated with increased mortality and morbidity in patients undergoing cardiac surgery (Koch et al., 2008; van Straten et al., 2010), which is another argument to make effort to limit blood trauma.

There are several phenomena contributing to the hemolysis in ECC. One of the main factors leading to blood cells destruction during ECC is oxidative stress. The source of reactive oxygen species (ROS) are the phagocytes (mainly neutrophils and monocytes) (Boyle et al., 1997). Their activation results in a respiratory burst, which initiates oxidation of cell proteins and plasma membrane lipids and in consequence causes an inactivation of enzymes, cell membrane depolarization, changes in plasma membrane fluidity and permeability (Matés et al., 1999; Kirkham and Rahman, 2006). Another of the important factors responsible for enhanced RBC destruction during ECC is the altered hemodynamic conditions. Mechanical stress may lead to the complete destruction of the RBC. Depending on how big the stress is, how long it acts, and what the RBC condition is, hemolysis may occur immediately or with delay (Kameneva et al., 2004; Cabrales, 2007; Grygorczyk and Orlov, 2017). Non-physiological flow conditions cause a decrease in RBC deformability and membrane potential, promoting a high blood viscosity. Turbulent flow, cavitation, inappropriate flow rates, decreased oncotic pressure caused by dilution of plasma, and the absence of flow pulsatility also directly contribute to RBC destruction in ECC systems (Ji and Undar, 2006).

So far, methods reducing ECC side effects were focused on perfusion sets miniaturization, improvement of the biocompatibility of the materials that have direct contact with blood, and optimization of hemodynamic conditions generated by the device. Unfortunately, the results obtained are still insufficient, as evidenced by the numerous complications occurring both during and after the ECC.

Our method was based on beneficial effects of low-level light therapy (LLLT) in the red-to-near-infrared radiation (R/NIR) spectral region on blood cells, with special regard to erythrocytes. It was shown that direct blood photobiomodulation cause alterations of the membrane fluidity and membrane potential which results in RBC aggregability decrease (Komorowska et al., 2002; Korolevich et al., 2004; Mi et al., 2004; Chludzińska et al., 2005), increased cell membrane mechanical resistance (Itoh et al., 2000; Komorowska et al., 2002; Chludzińska et al., 2005), and it may act protectively against oxidative stress (Chludzińska et al., 2005). During the exposure of RBCs to R/NIR light, dehydration process induces the photochemical dissociation oxyhemoglobin to deoxyhemoglobin and decreases the amount of physiologically inactive methemoglobin (MetHgb) (Walski et al., 2015).

The aim of this study was to evaluate the effect of LLLT applied during the ECC on the RBCs in an animal model.

## Materials and Methods

### Animals and Experimental Design

The study protocol was approved by a local II Ethical Review Board in Wrocław (II Lokalna Komisja Etyczna we Wrocławiu, approval No. 9/2013). Each animal was provided humane conditions and care according to the European Directive 2010/63/EU on the protection of animals used for scientific purposes.

The experiments were conducted on 24 clinically healthy female Polish Landrace pigs aged 5 months (mean weight 94.3 ± 3.2 kg) purchased from a single farm (The National Research Institute of Animal Production, Experimental Station in Pawłowice, Poland). The study was performed only in female pigs to preclude the impact of gender on the severity of hemolysis and oxidative stress (Kanias et al., 2016; Kander et al., 2017). After 1-week acclimatization period in the pens of the institutional vivarium, animals were randomly assigned into two experimental groups undergoing 1-h venoarterial ECC under general anesthesia: the control group and the LLLT group, in which the blood flowing through the oxygenator was exposed to R/NIR light. After decannulation, the animals were weaned from the ECC and transferred to the observation room for the following 23 h. During this time, several blood samples were collected to monitor the effects of ECC and R/NIR irradiation. At the end of the experiment, pigs were euthanized.

### Extracorporeal Circulation Procedure

Animals were anesthetized with intramuscular injection of ketamine (10 mg/kg, Bioketan, Vetoquinol Biowet Puławy, Poland), dexmedetomidine (10 μg/kg, Dexdor, Orion, Espoo, Finland) and diazepam (20 mg/100 kg, Relanium, Polpharma SA, Poland). Pigs were intubated and connected to a respirator (Wowo 500 Veterinary Anesthesia Machine, China). Mechanical ventilation was used in case of respiratory arrest lasting over 1 min or PCO_2_ in the exhaust phase exceeding 60 mmHg. Anesthesia was maintained with continuous rate infusion of propofol (0.2 mg/kg/min, Scanofol, ScanVet, Poland), fentanyl (50 μg/kg/10 min, Polfa SA, Poland), ketamine (10 mg/kg/30 min, IV), and diazepam (0.2 mg/kg/30 min, IV).

Prior to the ECC, a central venous catheter (11F/20 cm, dual-lumen polyurethane catheter, Medcomp^®^, United States) was inserted into the jugular vein to provide vascular access facilitating collection of subsequent blood samples scheduled for the experiment and drug administration. Jugular vein and carotid artery at the opposite side were surgically exposed and then cannulated with the ECC cannulas, which were connected to the drains of the perfusion set primed with 1500 ml crystalloid solution and 3000 IU unfractionated heparin (heparinum WZF^®^, Polfa Warszawa, Poland). When activated clotting time (ACT) was shorter than 200 s, additional doses of heparin were given to prevent extracorporeal blood clotting in the perfusion set. A single 1-h normothermic ECC was performed using Stoeckert 41-40-50 heart-lung machine (Sorin Group, Milan, Italy) with a roller pump and perfusion set Maquet VKMO 78000 (Maquet, Rastatt, Germany) in each animal. To complete the ECC set, an oxygenator Quadrox-i Adult, aortic filter, and a Dual Heater Cooler HCU-20 water pump (Maquet, Rastatt, Germany) were used. Main pump expenditure was maintained at 40–50% of the cardiac output and the air/oxygen mixer setting was 55%, with an oxygen flow of 2–3 L/min. Normal perfusion pressure and blood saturation were achieved in all animals. After weaning from ECC protamine sulfate was administered intravenously (Prosulf, Wockhardt, United Kingdom; 0.8 mg for every 100 units of previously given heparin) to normalize ACT. Afterward, the vascular cannulas were removed and the vessels were closed with an absorbable suture (4.0 PDS II, Ethicon LTD, Livingston, United Kingdom). The surgical wound was closed in two layers.

### Extracorporeal Blood Low-Level Light Treatment

A module for blood irradiation during ECC was installed on the oxygenator. Light emitter consisted of two non-coherent sources of radiation each built of low-voltage halogen burners with IRR coating in glass reflector, an optical diffuser, and NIR filter (Schneider-Kreuznach NIR IFG 098, Germany). Finally, non-polarized R/NIR electromagnetic waves in the range 750÷1500 nm with a maximum intensity of 800 nm illuminated the inlet and outlet chambers of the oxygenator. The blood flowing through the oxygenator was exposed to an average irradiance of 12.8 mW/cm^2^ throughout the whole ECC procedure. The dose of R/NIR radiation absorbed by the blood was 1.0 J/cm^3^.

### Blood Samples

**FIGURE 1:**
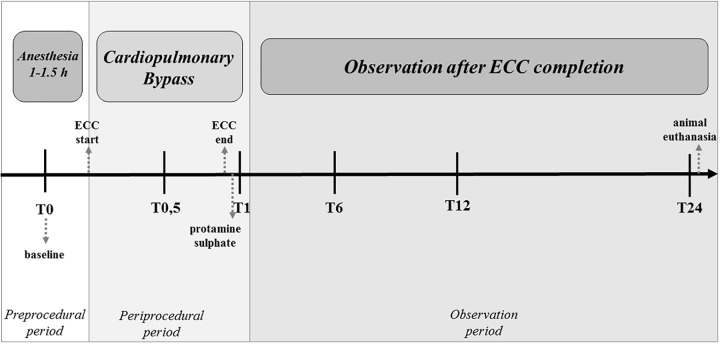
Schedule of blood sampling during the experiment. T0 – before commencement of ECC (baseline), T0.5 – 30 min after ECC initiation, T1 – directly after 1 h of ECC, protamine sulfate infusion and normalization of the ACT, T6 – 6 h after completing ECC treatment, T12 – 12 h after initiation of ECC, and T24 – 24 h after initiation of ECC.

Depending on the type of assays, venous blood was collected into test tubes containing 3.8% sodium citrate (FL Medical s.r.l., Torreglia, Italy) in a ratio of 1: 9 (anticoagulant: blood), K_2_EDTA (Profilab sc, Warsaw, Poland), heparin or with poly(methyl methacrylate) for serum preparation (FL Medical s.r.l., Torreglia, Italy). The blood was withdrawn immediately before the surgery, after 30 min of ECC, directly after completion of ECC and protamine sulfate infusion as well as in the post-ECC period: in the 6th, 12th, and 24th hour of the experiment (after ECC initiation) (**Figure 1**).

### Blood Analyses

Blood morphology measurements were performed using an automated hematology analyzer Scil Vet abc (Horiba, Kyoto, Japan) in K_2_EDTA blood. Serum lactate dehydrogenase activity (LDH) and bilirubin were measured spectrophotometrically using a commercial reagent (Alpha Diagnostics, Warsaw, Poland).

### Plasma-Free Hemoglobin Measurement

Plasma was obtained by centrifugation of citrated blood for 10 min at 1750 ×*g*. Plasma free Hgb (PFHgb) was quantified in each sample through colorimetric assay using the Drabkin’s reagent (Aqua-Med., Łódź, Poland). Each sample was measured in triplicate.

### Peroxidation of Red Blood Cell Membrane Lipids

Lipid peroxidation was measured as the amount of malondialdehyde (MDA) determined by the thiobarbituric acid reactive substance (TBARS) (Heath and Packer, 1968) with little modification described in detail elsewhere (Oleszko et al., 2015). Trichloracetic acid [TCA, Chempur, Poland; 15% (wt/vol) TCA in 0.25 M HCl] and thiobarbituric acid [TBA, AppliChem, Germany; 0,37% (wt/vol) TBA in 0.25 M HCl] were used. Equal volumes of RBC suspension, TBA, and TCA were mixed together. Samples enclosed in small glass tubes were heated at 100°C for 15 min, then cooled and centrifuged for 10 min at 1750 ×*g*. The absorbance of the supernatant was measured spectrophotometrically (Nicolet Evolution 60, Thermo Fisher Scientific, Waltham, MA, United States) at 535 nm and corrected for non-specific turbidity by subtracting the absorbance of the same sample at 600 nm. The content of TBARS was calculated using the extinction coefficient (155 mM^-1^ cm^-1^).

### Total Antioxidant Capacity

Total antioxidant capacity (TAC) was measured in plasma obtained by centrifugation of citrated blood as described by Re et al., 1999. Briefly, the method utilizes reduction of the bluish-green 2,2^′^-azinobis-(3-ethylbenzothiazoline-6-sulfonic acid) radical cation (ABTS^•+^) to colorless ABTS by the antioxidants present in the sample. The extent of decolorization by the antioxidants is determined as a function of concentration and time and calculated relative to the reactivity of Trolox as a standard. Stable ABTS^+^ solution was prepared by dissolving 19.5 mg of ABTS in 7 ml of PBS and adding 3.3 mg of potassium persulfate. Before each measurement, ABTS^•+^ solution was diluted in PBS so that its absorbance at 734 nm wavelength was equal to 1.0. A total of 5 μl of the sample was added to 995 μl diluted ABTS^•+^ and mixed for 6 min at 500 rpm in 37°C. The absorbance was measured at λ = 734 nm and the value of TAC was calculated from the standard curve.

### Haptoglobin Concentration

The Hp was measured spectrophotometrically in 10 times diluted blood plasma according to the method of Jones and Mould (1984) adjusted for microplate reader. The method is based on the detection of peroxidase-like activity of the haptoglobin-methemoglobin complex (Hp-MetHgb). The amount of hydrogen peroxide reduced by the Hp-MetHgb is proportional to the concentration of Hp. Breaking down of hydrogen peroxide leads to the oxidation of colorless guaiacol into yellowish-brown tetraguaiacol, which absorbance is measured at 492 nm.

### Statistical Analysis

**FIGURE 2:**
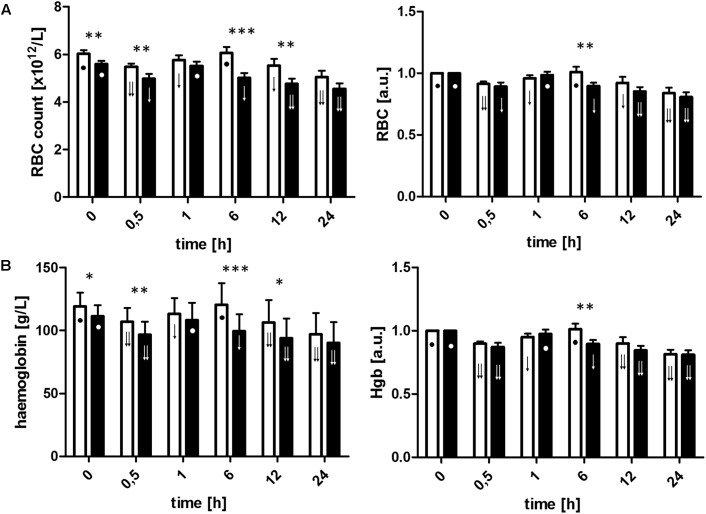
Red blood cell count **(A)** and hemoglobin concentration **(B)** calculated from raw data and relative values of the baseline, before commencement of ECC (T0), 30 min after initiation of ECC (T0.5), post-circulation and normalization of ACT (T1), and 6 h (T6), 12 h (T12), and 24 h (T24) after commencement of ECC in the control group (□) and LLLT (▪). Data are expressed as the mean (SD) (*n* = 12/group). ^∗^*p* < 0.05, ^∗∗^*p* < 0.01, ^∗∗∗^*p* < 0.001, LLLT group compared with the control group at the sampling time. Changes in the values of the measurement points in a given group during the experiment were denoted by: ↓↓, ↓, •. Values marked with the same symbol do not differ significantly. The analysis was performed at confidence level *p* < 0.05.

Longitudinal comparisons within groups were performed using bootstrap analysis of variance as described previously (Krishna Reddy et al., 2010; Walski et al., 2015). To test for differences between the groups at particular time points, the bootstrap *t*-test was used. The data were reported as a mean and standard deviation (SD). Although the groups were initially formed by random assignment, there were some differences in the RBC count and Hgb concentration at the baseline, so the results were presented as mean (SD) calculated from raw data as well as relative values to indicate the pattern of changes during experimental period (**Figures 2A,B** and **Table 1**). The results were considered significant for *p* < 0.05. Figures were performed using GraphPad^1^ Prism version 5 for Windows (GraphPad Software, San Diego, CA, United States).

**Table 1:** Hematological and biochemical parameters assessed during entire experimental period.

Parameter	Group	T0		T0.5		T1		T6		T12		T24		Reference ranges
		Mean	*SD*	Mean	*SD*	Mean	*SD*	Mean	*SD*	Mean	*SD*	Mean	*SD*	
RBC count	CTR	6.02	0.49	5.49	0.43	5.78	0.63	6.06	0.83	5.53	0.91	5.05	0.85	5.00 ÷ 8.00
(× 10^12^/L)	NIR	5.60	0.41	4.99<	0.60	5.51	0.56	5.02	0.61	4.77<	0.72	4.54<	0.84	
Hgb	CTR	119	11	107	11	113	12	120	16	107	17	97	16	100 ÷ 160
(g/L)	NIR	111	9	97^<^	10	108	13	100	13	94^<^	15	90^<^	16	
PFHgb	CTR	1.89	0.49	1.30	0.43	1.81	0.41	1.67	0.54	1.31	0.35	1.58	0.35	0.81 ÷ 2.9^a^
(mg/L)	NIR	1.83	0.50	0.96	0.29	1.33	0.43	1.04	0.34	0.95	0.34	1.17	0.47	
Bilirubin	CTR	4.25	1.90	–	–	5.47	1.90	3.83	1.51	4.22	2.61	3.36	1.00	0.00 ÷ 17.10
(μmol/L)	NIR	4.39	1.50			3.66	1.18	3.25	0.92	2.99	1.26	3.72	1.23	
Haptoglobin	CTR	0.49	0.50	0.31<	0.42	0.31<	0.37	0.29<	0.42	0.35<	0.35	1.01	0.23	0.37 ÷ 0.69^a^
(g/L)	NIR	0.57	0.49	0.44	0.46	0.37	0.44	0.38	0.42	0.61	0.41	1.26	0.16	
LDH	CTR	421	159	407	153	481	155	872^>^	370	735^>^	404	931^>^	840	380 ÷ 634
(U/L)	NIR	526	73	435	98	560	127	616	180	608	117	524	89	
WBC count	CTR	16.02	4.63	12.54	5.66	14.13	6.82	22.38>	5.29	19.43	5.76	16.54	4.75	11.0 ÷ 22.0
(× 10^9^/L)	NIR	18.63	5.21	16.38	7.16	16.60	6.25	21.66	5.68	20.30	5.25	15.56	5.60	
TBARS	CTR	11.80	1.73	14.48	2.94	14.14	2.60	17.01>	7.68	16.01>	7.68	15.78>	6.01	7.79 ÷ 15.72^a^
(nmol/gHgb)	NIR	11.72	2.02	12.28	2.26	10.58	1.47	14.31	3.51	14.58	3.42	13.42	3.63	
TAC	CTR	1.60	0.06	1.37	0.08	1.50	0.09	1.53	0.09	1.61	0.12	1.60	0.08	1.40 ÷ 1.77^a^
(mmol Trolox eq./L)	NIR	1.57	0.11	1.39	0.11	1.48	0.12	1.56	0.15	1.58	0.11	1.61	0.11	


*^a^Reference range estimated by the authors using baseline values. ^>^Mean value above a reference range. ^<^Mean value below a reference range.*

### Analysis of the Reference Ranges

All the available reference ranges for pigs were from Latimer et al. (1981). Although there are no reference ranges for pigs for PFHgb, Hp, TBARS, and TAC, the 95% reference intervals for these parameters were acquired using baseline values (T0). When examined values showed a normal distribution in the Kolmogorov–Smirnov test, we acquired a 95% interval as the reference interval. When examined values did not show a normal distribution, a bootstrap methodology was applied (Horn et al., 1998; Horn and Pesce, 2003).

## Results

### Animals

All animals survived the procedure of 1-h normothermic ECC and the 23-h observation period and remained in a good clinical condition during the entire experiment. Each pig was euthanized after the observation period. No technical problems with the ECC occurred during the experiment.

### Red Blood Cells

The ECC initiation was associated with hemodilution which directly contributed to a significant drop in measured parameters at T0.5 (**Figures 2A,B** and **Table 1**). Both the RBC count and Hgb concentration decreased similarly in both groups throughout the ECC. Although in both groups postoperative changes in RBC count and Hgb showed similar trends – after initial return to baseline value constant drop in both parameters was observed – the time point when the values returned to the preoperative was shifted in time when compared between groups (in LLLT group it was observed directly after ECC while in the control group just in the 6th hour of the experiment). It resulted in the biggest difference of parameters value between groups at T6 – significantly lower values were measured in LLLT group (5.02 × 10^12^/L vs. 6.06 × 10^12^/L, *p* < 0.001, and 85 g/L vs. 102 g/L, *p* < 0.001, for RBC count and Hgb concentration, respectively). At the end of observation, both parameters were found to be comparable in study and control group. One of the possible causes associated with the decrease in RBC and Hgb during the study could be a relatively high volume of blood samples (c.a. 120 ml at each time point).

### Hemolysis Parameters

**FIGURE 3:**
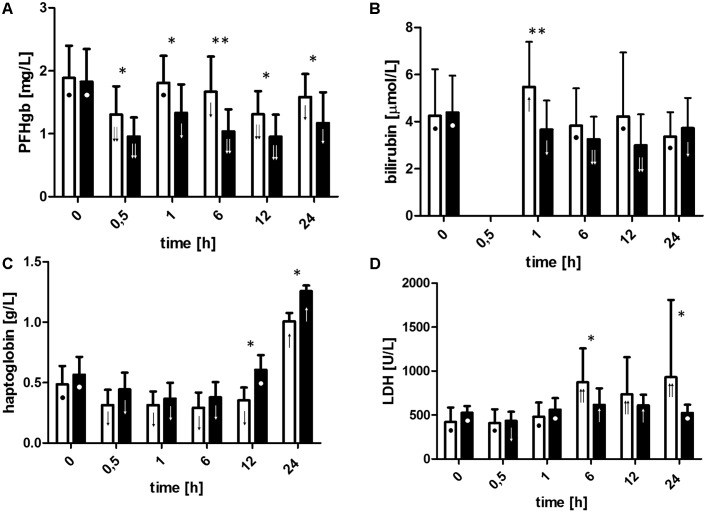
Changes in plasma-free hemoglobin **(A)**, bilirubin **(B)**, haptoglobin **(C)**, and lactate dehydrogenase level **(D)**. For definition of time points, see **Figure 2** caption. Data are expressed as the mean (SD) (*n* = 12/group). ^∗^*p* < 0.05, ^∗∗^*p* < 0.01, ^∗∗∗^*p* < 0.001, LLLT group compared with the control group at the sampling time. Changes in the values of the measurement points in a given group during the experiment were denoted by: ↓↓, ↓, •, ↑, ↑↑. Values marked with the same symbol do not differ significantly. The analysis was performed at confidence level *p* < 0.05.

The courses of PFHgb and bilirubin verified significant advantages in favor of LLLT (**Figures 3A,B**). The level of PFHgb decreased significantly after initiation of the ECC (p < 0.05) both in the control and in the LLLT groups. However, the decrease was significantly higher in the LLLT group (drop to the concentration of 0.10 g/L vs. 0.13 g/L, *p* < 0.05). Hemolysis increased after weaning from the ECC (rise in the level of PFHgb compared to T0.5). In the LLLT group in the observation period, both parameters were lower than before ECC and moreover lower than in the control group (levels of PFHgb in all postoperative time points, bilirubin at T1). Cell-free Hgb is released into the circulation in subjects undergoing ECC, so Hp showed the expected drop because of the binding of PFHgb (**Figure 3C**).

Not only PFHgb and serum bilirubin concentration but also serum LDH activity showed differences between the groups in favor of LLLT (**Figure 3D**). The activity of LDH increased significantly during the observation period (*p* < 0.05 for all) in the control group whereas in the LLLT group after an initial increase at T6 and T12 it was normalized at T24. In all time points (except baseline), LDH activity was higher in the control group compared to the LLLT group. No statistically significant differences between the groups were observed during the ECC, however, when the activity of LDH in the LLLT group decreased presumably due to hemodilution (T0.5 vs. T0, *p* < 0.05), whereas in the control group no drop in serum LDH activity was detected despite identical changes in plasma volume. Moreover, significantly lower serum LDH activity values in the LLLT group clearly demonstrate the persistence of LLLT effects during the observation period.

### Inflammation and Oxidative Stress Markers

**FIGURE 4:**
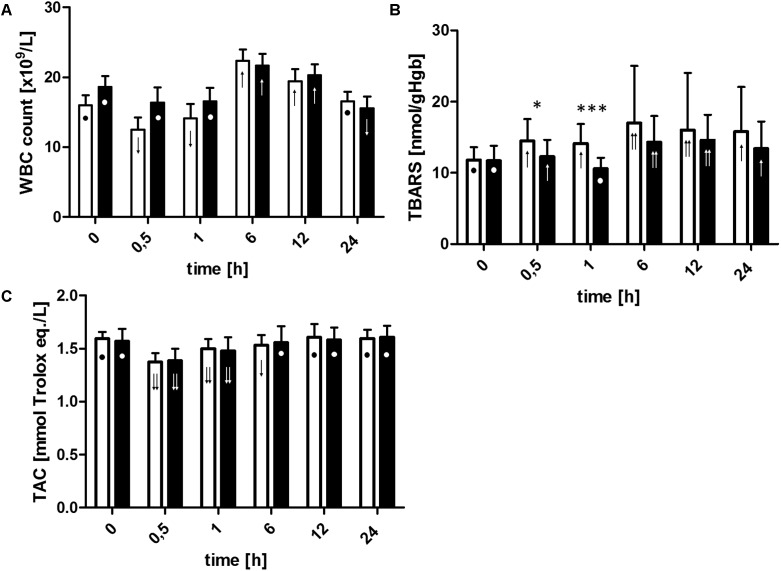
Changes in White Blood Cell count **(A)**, thiobarbituric acid reactive substances concentration **(B)**, and plasma total antioxidant capacity **(C)**. For definition of time points, see **Figure 2** caption. Data are expressed as the mean (SD) (*n* = 12/group). ^∗^*p* < 0.05, ^∗∗∗^*p* < 0.01, LLLT group compared with the control group at the sampling time. Changes in the values of the measurement points in a given group during the experiment were denoted by: ↓↓, ↓, •, ↑, ↑↑. Values marked with the same symbol do not differ significantly. The analysis was performed at confidence level *p* < 0.05.

The ECC initiation resulted in white blood cells (WBCs) count decrease in the control group. Afterward, the values slowly increased until the end of the ECC (**Figure 4A**). In contrast, WBC count was constant during whole ECC procedure when blood photobiomodulation was applied. A sharp increase in the number of WBC released into the peripheral blood at the 6th hour of the experiment and a fall to the baseline level (control group) or below baseline level (LLLT group) at the end of observation were noted. The patterns of WBC count changes were similar for both groups, however, the growth rate between the end of ECC (T1) and the peak value (T6) in the LLLT group was significantly lower when relative values are taken into an account (21.66 × 10^9^/L vs. 22.38 × 10^9^/L, ns. at T6, or increase by 30% vs. 58% at T6 in relation to T1, *p* < 0.05, for LLLT and control groups, respectively).

The concentrations of the TBARS increased significantly (for all time points, except for T1) in the control group with the peak at 6th hour post-circulation (17.01 nmol/gHgb vs. 11.80 nmol/gHgb when compared to the T0 value) and remained elevated over the upper reference limit throughout the whole observation period, while in the LLLT group RBC membrane lipid peroxidation slightly increased at T0.5 (12.28 nmol/L vs. 11.72 nmol/L when compared to the T0 value), returned to the baseline concentration at the end of ECC, furthermore, reached the maximum level at 12th hour (14.58 nmol/L), and finally declined to a moderately higher concentration than the baseline value (13.42 nmol/L) at the end of observation period (all described changes were significant at *p* < 0.05) (**Figure 4B**). TBARS concentration was significantly lower in the LLLT group during the whole ECC (*p* < 0.05 at T0.5, *p* < 0.001 at T1, with respect to the control group).

The ECC initiation was associated with TAC significant drop in both groups and gradual increase of the measured parameter up to the baseline achieved after 12 h of observation in the control group and after 6 h in the LLLT group, however, no significant differences between the groups were noted (**Figure 4C**).

## Discussion

To the best of our knowledge, our experiment is a first *in vivo* study which demonstrates that LLLT using R/NIR radiation may provide support for therapeutic procedures using ECC by limiting side effects associated with the activation and damage of extravasated blood cells.

During ECC, the primary cause of inflammation is blood contact with the non-physiological, polymer surfaces of the ECC system. Almost immediately after the beginning of ECC, plasma proteins are adsorbed on the biomaterials of ECC system. This coat consists mostly of fibrinogen and albumin, however, the content of individual proteins depends on their concentration in patients’ plasma and the properties of biomaterial (Edmunds, 1995). Due to the conformational changes of the adhered proteins, receptors for circulating plasma proteins and blood cells are exposed. Finally, the activation of plasma cascade systems, endothelial cells, platelets, and leukocytes occurs (Edmunds, 1998).

Inflammatory response and oxidative stress are inherent disorders associated with ECC (Kawahito et al., 2000; Warren et al., 2009; Mamikonian et al., 2014; Zakkar et al., 2015), what was further confirmed by the results of our study. In the control group, a decrease in WBC count during ECC was accompanied by a significant increase in TBARS concentration. These changes result from two overlapping processes. On one hand, ECC is associated with hemodilution, on the other hand, neutrophils are activated by the contact with biomaterial and form platelet-leukocyte aggregates or they are sequestrated in the pulmonary vasculature as well as marginated to the tissues (mainly lungs and heart). In contrary, in the LLLT group, both WBC count and TBARS concentration remained relatively constant during ECC. Since hemodilution in both groups was comparable, it may be assumed that LLLT reduced generation of ROS, which contribute to the damage of RBCs plasma membranes. Moreover, photobiomodulation resulted in a limitation of the inflammatory response in the observation period as also evidenced by the significantly lower C-reactive protein (CRP) concentration (c.a. 60% lower at T6, *p* < 0.05, data not shown) in the postoperative period.

The impact of LLLT on oxidative stress in our study is consistent with the literature reports. It was previously shown that *in vitro* blood exposition to LLLT diminished platelet adhesion and aggregation when subjected to shear stress on subendothelial extracellular matrix-coated plates in time-dependent manner. The inhibitory effect was reversible up to 1 h after the termination of irradiation (Brill et al., 2000). More recently, Rola et al. (2017) confirmed decreased whole blood aggregation after LLLT exposure by the mechanisms independent of the NO metabolism and without significant effect on the release of platelet activation markers. It is important because platelet activation due to contact with biomaterial of ECC or alternating shear stress results in P-selectin expression, which binds to P-selectin glycoprotein ligand-1 in the plasma membranes of leukocytes. Platelet-leukocyte aggregates cause leucotrienes and proinflammatory cytokines release, thus stimulating inflammatory response. The activated polymorphonuclear leukocytes (mainly neutrophils) are believed to be a prime source of ROS during cardiac surgery, leading to peroxidation of erythrocytes plasma membrane lipids (Kamath et al., 2001; Zakkar et al., 2015). It can be concluded that the benefit of reversible inactivation of thrombocytes by LLLT may be a reduction of oxidative stress. However, direct suppression of the human neutrophils ROS production by NIR radiation was demonstrated by Shiraishi et al. (1999) and Fujimaki et al. (2003). Concomitantly, the serum opsonic activity was decreased, and this suppressive effect might be caused by inhibiting the activation of the classical and alternative complement pathway (Shiraishi et al., 1999). Since the primary mechanism of LLLT action is attributed to changes in hydrogen bond energy after absorption of R/NIR radiation and in consequence water molecules dissociation, that was firstly described by Goodall and Greenhow (1971), resulting in the mild conformational changes, the extent of the observed LLLT effects may be very broad (Goodall and Greenhow, 1971; Knight et al., 1979; Natzle et al., 1981; Walski et al., 2015). However, it is also important that the impact of LLLT during ECC persisted in the observation period limiting propagation of the inflammatory response, what was reflected in a significantly lower WBC count. In similar experimental models, neutrophil-free radical production was elevated 6 h after CPB compared to pre-CPB levels, accompanied by the highest neutrophil count, plasma TNF-α, IL-6, and IL-8 concentrations (Schwartz et al., 1998; Kawahito et al., 2000).

As a consequence of increased oxidative stress and hemodilution, a decrease in TAC was recorded. In the course of the ECC TAC dropped at a similar rate in both groups. As depicted in **Figure 4C**, the TAC values recovered initial levels during the postoperative period at 6th hour in LLLT group and at 12th hour in control group. Changes in TAC during CPB procedures have been widely discussed (Toivonen and Ahotupa, 1994; Clermont et al., 2002; Hadjinikolaou et al., 2003; Luyten et al., 2005). However, due to conflicting results, it was not possible to clearly demonstrate the mechanism responsible for TAC changes during and post ECC. In our opinion, initially, decrease of TAC during ECC is an indicator of a response to increased oxidative stress. Thus, to provide oxidative balance, scavengers of ROS are involved and utilized and till the new reserve of antioxidants is supplied, TAC is temporarily decreased. TAC return to baseline level is probably caused by a release (synthesis) of antioxidants into the blood plasma related to production of acute phase proteins (e.g., Hp, ceruloplasmin) and/or release of intracellular antioxidants (e.g., superoxide dismutase, catalase, glutathione, uric acid) from damaged cells (Hadjinikolaou et al., 2003).

Another consequence of ECC is morphotic blood elements damage resulting from their exposition to non-physiological mechanical (e.g., inappropriate flow rates, turbulence, cavitation) and environmental (e.g., decreased oncotic pressure by hemodilution, hypothermia) factors. It is manifested by immediate and delayed hemolysis and by changes in the mechanical properties of RBCs (Vercaemst, 2008; Olia et al., 2016). It is noteworthy that RBCs are much more resistant to mechanical stress than platelets and WBCs, and therefore, it is assumed that once RBCs are damaged, other blood elements have already been affected more severely (Vercaemst, 2008). The present study showed that LLLT using R/NIR radiation is capable to reduce RBC damage induced by ECC. In our experimental model, we were not able to see a severe hemolysis, as it is often the case during ECC procedures conducted in patients, in whom additional RBCs traumatization is related to surgery and ischemia-reperfusion episode (CPB with cardiac arrest and aortic clamping) (Pierangeli et al., 2001; Mamikonian et al., 2014). Although an increasing concentration of PFHgb always indicates hemolysis, normal values are not a necessary indicator of a healthy RBC (Vercaemst, 2008). Hemolysis associated with cardiovascular/circulation assist devices is assessed on the basis of criteria approved by the Interagency Registry for Mechanically Assisted Circulatory Support (INTERMACS). According to them, a minor hemolysis occurs when PFHgb concentration is greater than 20 mg/dl or LDH activity is greater than 2.5× the upper limit of the normal range (INTERMACS, 2016). In our experiment, none of the measured parameters exceeded the INTERMACS criteria, nevertheless, results of our study indicated the presence of blood trauma induced by ECC more severely in the control group. In the LLLT group, PFHgb concentration was significantly lower at each time point (except baseline) when compared to control group. Changes in LDH activity were more explicit because in the control group serum LDH activity strongly increased starting from T6 when it exceeded the upper limit of the reference range and remained elevated until the end of the experimental period. In contrast, in LLLT group LDH values were significantly lower in the observation period. Hp late increase might be associated with an inflammatory reaction as it acts as a moderate acute-phase protein, however, when blood is hemolytic, determination by Hgb binding assays may give unreliable results (Gruys et al., 2005). Our hemolytic parameters measurements are quite consistent with the results obtained by others (Cirri et al., 2001; Mamikonian et al., 2014; Vermeulen Windsant et al., 2014; Baumbach et al., 2016). Sublethal RBC damage results in decreased deformability and surface charge, and increased fragility and aggregability (Lee et al., 2007; Vercaemst, 2008) which subsequently lead to the early removal of the RBC from the circulation by the spleen or in the altered rheological proprieties of the blood followed by compromising efficient microcirculation and oxygen delivery to the surrounding tissues (Watanabe et al., 2007; Vercaemst, 2008; Ciana et al., 2017). Further blood trauma results in lethal damage of the ruptured, overstretched, or prematurely aged RBCs indicated by the intravascular hemolysis development (Olia et al., 2016). However, excessive increase in PFHb does not occur until Hp and NO scavenging capability is saturated. Even mild or transient hemolysis may provoke serious complications like thrombosis, postoperative kidney injury, and smooth muscle dystonia, vasculopathy, or endothelial dysfunction (Rother et al., 2005; Vercaemst, 2008; Vermeulen Windsant et al., 2014; Olia et al., 2016).

As we showed, LLLT application during ECC may significantly reduce the RBCs damage and thus limit hemolysis-related complications. Protective photochemical effect of R/NIR radiation on RBCs *in vitro* was demonstrated by Itoh et al. (1996, 2000) on ECC model. During the 4-h experiment, a dramatic decrease in erythrocytes intracellular ATP and deformability accompanied by the intense hemolysis was detected. LLLT irradiation limited the destructive impact of ECC on RBCs concomitantly, no significant difference was observed in the partial oxygen pressure, between the LLLT and control groups, indicating that erythrocyte oxygenation was maintained throughout the experiment. Moreover, SEM of the oxygenator membranes revealed a significantly higher number of discocytes in the laser treated sample compared with control (45 and 20%, respectively) (Itoh et al., 1996, 2000). Decreased hemolysis and reduced plasma lipid peroxidation resulting from exposure of erythrocytes to R/NIR radiation have also been demonstrated in experiments, in which samples pretreated with R/NIR radiation (700–2000 nm) were ozonated (Komorowska et al., 2002; Chludzińska et al., 2005). Changes in RBC osmotic fragility caused by R/NIR radiation also indicate plasma membrane stabilization and decreased hemolysis in the hypotonic environment (Iijima et al., 1991; Habodaszova et al., 2004). Furthermore, Walski et al. (2014) showed that osmotic properties of human erythrocytes subjected to NIR radiation are unified, what manifests as a change in slope of the hemolysis curve. Narrowing of population distribution suggests the strongest impact of NIR radiation on the most and least resistant cells.

An improvement of RBCs mechanical properties under the influence of LLLT using R/NIR may be explained by changes at the cellular and subcellular levels, mainly by an increase in overall activity of ATP-ases, especially sodium-potassium ATP-ase (Kujawa et al., 2004; Pasternak et al., 2012), which maintains resting membrane potential and is responsible for cell volume regulation (Lew and Tiffert, 2017). There are two mechanisms which explain changes in enzyme activity after R/NIR radiation: conformational changes in the protein structure, and indirect modification of the surrounding molecules. It has been shown that R/NIR radiation induces structural changes in the lipid bilayer. It causes a decrease in polarity of phospholipid hydrophilic fragments. Moreover, lower doses of LLLT diminished the order parameter, which is a measure of the relative fluidity in the membranes (Komorowska and Czyżewska, 1997). Higher RBC membrane fluidity after exposure to NIR radiation has also been confirmed in other studies (Kujawa et al., 2003; Chludzińska et al., 2005) and its macroscopic consequence is an improvement of the blood rheological properties related with increased erythrocytes deformability, which was observed both *in vitro* and *in vivo* (Iijima et al., 1993; Mi et al., 2004; Wang et al., 2016).

Furthermore, it has been demonstrated that R/NIR radiation causes an increase in electrokinetic potential and therefore may directly inhibit RBCs aggregation (Komorowska et al., 2002; Mi et al., 2004; Korolevich et al., 2004; Chludzińska et al., 2005). Erythrocytes are characterized by a tendency to form rouleaux and aggregates, thus influencing blood rheology (Steffen et al., 2011; Flormann et al., 2017). Pathological conditions such as cardiovascular disease (CVD) are accompanied by a presence of larger RBCs aggregates and a relative decrease in a number of singular erythrocytes. While in healthy individuals rouleaux are seen, in patients with CVD RBC agglutination occurs and irregular erythrocyte aggregates with a diameter up to 300 μm may be found (Korolevich et al., 2004; Brust et al., 2014; Flormann et al., 2017). The 5–15 min LLLT of the temporal region increased local microcirculation (measured as perfusion rate) almost twofold and caused a change in the aggregation characteristics of RBCs (Korolevich et al., 2004).

However, an improvement in microcirculation may result also from blood vessel relaxation in response to NO. It has been repeatedly reported, that R/NIR radiation leads to a partial photochemical dissociation of Hgb-ligand complexes (e.g., O_2_, CO, NO) during radiation absorption (Komorowska et al., 2002; Vladimirov et al., 2004; Zalesskaya and Sambor, 2005; Walski et al., 2015). Moreover, Hgb deoxygenation causes tyrosine phosphorylation in band 3 protein. It results in its stronger binding with spectrin network of RBC. Erythrocyte membrane mechanical resistance increases and the rate of hemolysis decreases, suggesting greater resistivity to osmotic stress (Chludzińska et al., 2005).

Another important issue is the influence of photodynamic reactions induced by LLLT on the ability of blood to transport oxygen. On one hand, erythrocytes *in vitro* exposed to R/NIR light demonstrate a rapid increase of oxygen saturation (SpO_2_) of Hgb and an increase of oxygen tension in the blood whereas both PaCO_2_ and pH shows no significant changes after irradiation (Wąsik et al., 2007). An increase of oxy-Hgb concentration related with LLLT was also observed *in vivo* in humans during placebo-controlled studies (Tian et al., 2016; Wang et al., 2016). On the other hand, Yesman et al. (2016) observed the reduction in SpO_2_ up to 5% which indicated the process of photodissociation of HbO_2_ *in vivo*. However, despite the contradictory results, the final outcome of each study was an improvement of the tissue oxygenation as the LLLT can promote the release of oxygen from oxy-Hgb (Asimov et al., 2006; Tian et al., 2016; Wang et al., 2016; Yesman et al., 2016; Xu et al., 2018).

All in all, RBC modified by LLLT has a larger electrokinetic charge, higher plasma membrane fluidity and increased resistance to oxidative and mechanical stress. All these features may contribute to the reduction of hemolysis during ECC demonstrated in our study. Concomitantly, it is likely, that NO intravascular bioavailability increases after LLLT. Thus, the outcome in patients treated with ECC could theoretically be improved by LLLT as a factor at least partly eliminating toxic hemolysis-derived end products and rheological-altered blood cell characteristics. Although after the ECC hematocrit is not reduced only because of hemodilution or RBCs destruction but also as a result of erythropoiesis inhibition due to acute postoperative inflammation, LLLT application might accelerate erythropoiesis recovery and thus ensure a better outcome for patients undergoing cardiac surgery. However, this hypothesis should be validated in further studies which would include at least prolonged observation period.

### Limitation of the Study

Our CPB animal model was quite free from bias related to other surgical trauma. To investigate the effect of ECC on RBCs and oxidative stress, we used a normal animal model to rule out the effect of other pathological factors on the experimental results. Additionally, animals were subjected to ECC without cardiac arrest, aortic clamping, or any cardiac surgery. On one hand, it enabled us to analyze the results as the pure effect of ECC, on the other hand, other pathological factors which are present during cardiac surgery were omitted so it is difficult to assess what meaning the changes we observed would have in real-life patients. Moreover, 1-h ECC may be too short to provoke extensive hemolysis and RBC damage. The decrease in RBC we noted after 6th hour of the experiment may be at least partially due to repeated blood collection. And the last but not least – it was previously determined that porcine RBC are less fragile to shear stress than human RBC and may, therefore, underestimate the impact of the ECC on the blood components (Chan et al., 2017), what is a common challenge of research results translation from model organisms to human (Minetti et al., 2013).

## Conclusion

This study showed that ECC induces oxidative stress and blood cells destruction when performed in the porcine model of CPB even without ischemia/reperfusion injury or cardiac surgery. Our data clearly present that LLLT may significantly reduce blood trauma in this kind of treatment. The effect of extracorporeal blood photobiomodulation is both limited inflammatory response to ECC as well as RBCs damage and thus minimized hemolysis-related complications.

## Author Contributions

TW, AD, and MK defined the study and planned the experiments. AC, GW, and RC were responsible for the optimization of ECC protocol. TW, AD, JB, AC, GW, NT-P, and MG performed the acquisition and analysis. TW, AD, and MK interpreted the data. TW, AD, JB, and NT-P drafted the manuscript. AC, MK, and RC critically revised the manuscript. All authors approved the final version of the manuscript.

## Conflict of Interest Statement

Theauthors declare that the research was conducted in the absence of any commercial or financial relationships that could be construed as a potential conflict of interest.

## Footnotes

1 www.graphpad.com
